# Supplementary material for: MetaRibo-Seq measures translation in microbiomes
Source: Nat Commun. 2020 Jun 29;11:3268. doi: 10.1038/s41467-020-17081-z (PMC7324362; doi:10.1038/s41467-020-17081-z)
Supplement: Supplementary file 10 — Supplementary Data 7 [file 41467_2020_17081_MOESM10_ESM.zip › File2/Confidence_High_Taxonomy/18898_out.krona.html]

Javascript must be enabled to view this page.

members
magnitude
magnitudeUnassigned
count
unassigned
taxon
rank

18898\_out

2

2
2
superkingdom

phylum
1
976

1
200643
class

order
171549
1

171552
1
family

genus
838
1


SRS147541\_contig\_number\_contig-100\_5275.45687
species
28137
1

1
1224
phylum

subphylum
68525
1

28221
1
class

213115
1
order

194924
1
family

genus
35832
1


SRS098655\_contig\_number\_1127
species
1
35833
